# 397. Oral Vancomycin Versus Fidaxomicin for *Clostridiodes difficile*: A Comparative Retrospective Evaluation across a Large, Multi-center Healthcare System

**DOI:** 10.1093/ofid/ofac492.475

**Published:** 2022-12-15

**Authors:** Kaleb Roemer, Hayley Burgess, Kenneth E Sands, Ryan Hodgin

**Affiliations:** HCA Healthcare, Nashville, Tennessee; HCA Healthcare, Nashville, Tennessee; HCA Healthcare, Nashville, Tennessee; HCA Healthcare, Nashville, Tennessee

## Abstract

**Background:**

The Infectious Diseases Society of America and Society for Healthcare Epidemiology of America recently published the 2021 Focused Clinical Practice Guideline Update on Management of *Clostridioides difficile* in Adults that recommends fidaxomicin as a preferred treatment for initial and recurrent C. difficile infection (CDI) over oral vancomycin. The aim of this study was to evaluate clinical outcomes of adult inpatients with CDI diagnosis treated with fidaxomicin versus oral or rectal vancomycin.

**Methods:**

The patient population included adult inpatients 18 years or older admitted to a hospital within the healthcare system with CDI *International Classification of Diseases, Tenth Revision* (ICD-10) codes from January 1, 2017 to October 31, 2021. Data was obtained for inpatients with a discharge diagnosis of CDI and receiving fidaxomicin, oral or rectal vancomycin, or no receipt of either antibiotic. Readmission was defined as readmittance to the healthcare system within 8 weeks and a CDI diagnosis. Recurrence risk factors were defined as age 65 years or older, immunocompromised based on ICD-10 codes, and severe CDI defined as white blood cell count > 15 cells/mm^3^ PLUS serum creatinine > 1.5 mg/dL.

**Results:**

More than 45,000 inpatients with a CDI diagnosis were evaluated. Administration rates of vancomycin, fidaxomicin, or no receipt of either was 76%, 6% and 18%, respectively. Median length of stay for vancomycin compared to fidaxomicin was 7 versus 9 days, respectively. Readmission rate was lower for total inpatients receiving vancomycin (14.6%) compared to fidaxomicin (18.5%), showing statistical significance (p < 0.0000005). Subgroup analyses were performed to evaluate inpatients with risk factors for CDI recurrence. Vancomycin had a lower readmission rate for all recurrence risk factor groups. Additionally, oral vancomycin had lower readmission rates for patients with more than one risk factor (13.4% vs. 16.1%).

**Conclusion:**

The results of this study demonstrated that fidaxomicin did not result in a shorter length of stay or lower readmission rate compared to oral/rectal vancomycin. This study supports the continued use of oral/rectal vancomycin for CDI without preference for fidaxomicin.

**Disclosures:**

**All Authors**: No reported disclosures.

